# Tracking performance and its underlying characteristics in talented swimmers: a longitudinal study during the junior-to-senior transition

**DOI:** 10.3389/fphys.2023.1221567

**Published:** 2023-08-09

**Authors:** Aylin K. Post, Ruud H. Koning, Chris Visscher, Marije T. Elferink-Gemser

**Affiliations:** ^1^ Center for Human Movement Sciences, University Medical Center Groningen, University of Groningen, Groningen, Netherlands; ^2^ Department of Economics, Econometrics and Finance, Faculty of Economics and Business, University of Groningen, Groningen, Netherlands

**Keywords:** youth athletes, talent development, acquisition of expertise, competitive swimming, sports performance, longitudinal analysis, multidimensional approach

## Abstract

The present study strived to gain a more profound understanding of the distinctions in development between swimmers who are considered to be on track to the senior elite level compared to those who are not. Longitudinal data of 29 talented sprint and middle-distance swimmers (12 males; 17 females) on season best performances (season best times) and underlying performance characteristics (anthropometrics, starts, turns, maximal swimming velocity, stroke index [SI, an indirect measure of swimming efficiency] and lower body power) were collected over four swimming seasons (median of *n* = 3 seasons per swimmer). Based on their season best performance at early senior age (males aged 18–19; females aged 17–18), some swimmers were considered to be on track to reach the elite level (referred to as high-performing seniors; 6 males and 10 females), whereas others were not (referred to as lower-performing seniors; 6 males and 7 females). Retrospectively studying these swimmers (males and females separately), we found that all high-performing seniors were already on track to the elite level at late junior age (males aged 17; females aged 16), evidenced with faster season best performances throughout their transition compared to their lower-performing peers (*p* < 0.05). Independent sample *t*-tests revealed that high-performing seniors significantly outscored their lower-performing peers on maximal swimming velocity (males and females), starts and turns (males), SI (females) and lower body power (females) at late junior age (*p* < 0.05). Additionally, multilevel models showed faster rates of development for high-performing seniors on turns and maximal swimming velocity (males), and SI (females) compared to lower-performing peers during the junior-to-senior transition (*p* < 0.05). Particularly, the higher initial levels of swim performance and underlying characteristics at late junior age as well as the ability to keep progressing on season best performances (males and females), turns and maximal swimming velocity (males), and SI (females) during the junior-to-senior transition, may be crucial factors in the attainment of swimming expertise.

## 1 Introduction

Competitive swimming is a popular, global sport wherein the finest of margins can determine whether one attains the title or falls short (World Aquatics, 2021). The fastest swimmer is the one who sustains the greatest power output in an efficient and skillful manner throughout the event (Miyashita, 1996). This is influenced by a highly complex interaction of underlying performance characteristics such as anthropometrical (e.g., height), physiological (e.g., muscle power), technical (e.g., stroke index), tactical (e.g., pacing behaviour) and psychological (e.g., self-regulation of learning) factors (Barbosa et al., 2010; Saaverda et al., 2010). As a result, swimming performance is not defined by a fixed set of underlying performance characteristics, but rather achieved through individualistic combinations which can change throughout a swimmer’s career (Vaeyens et al., 2008; Elferink-Gemser & Visscher 2012; Barbosa et al., 2013; Barbosa et al., 2019).

While acknowledging that swimmers have unique profiles contributing to swimming performance, cross-sectional studies show a range of characteristics that set elite swimmers (i.e., those ranked in the top 50 worldwide) apart from non-elites. These include faster progression of swim performance between and within seasons (Post et al., 2020a; Post et al., 2020b); a highly efficient stroke (Sánchez & Arellano, 2002); pacing behaviour which better fits the tasks demands (Lopez-Belmonte et al., 2022; Menting et al., 2023) and advantageous anthropometrics (Rejman et al., 2018). However, with most studies in elite swimming focusing on adults (Costa et al., 2012), little is known about the developmental pathway towards swimming expertise. For example, the systematic narrative review of Morais et al. (2021) found only eight longitudinal studies on youth swimmers’ development over multiple seasons, highlighting the need for research that shed light on the journey towards swimming excellence.

In particular, research on the development of swim performance and its underlying characteristics during the junior-to-senior transition is lacking. This normative transition signifies the moment at which swimmers start to participate in adult competitions (Larsen and Alfermann, 2017), which is typically driven by age-related policies of a swimming federation. Apart from inherent changes in practice and competition, like competing in the open age category instead of annual age categories, the transition from junior to senior in sports frequently aligns with significant other life transitions, such as the move from high school to university (Wylleman and Lavellee, 2004). Consequently, the junior-to-senior transition is considered as the most demanding and difficult phase in the trajectory towards the elite level (Stambulova et al., 2009). During this critical stage, many talented athletes face stagnation, opt for recreational sports, or even discontinue their athletic pursuits, while only a select few master the transition to the senior elite level (Stambulova et al., 2009; Güllich et al., 2023). In swimming, this is exemplified by the study of Brustio et al. (2021) which found that the junior-to-senior transition rate amongst elite European swimming sprinters was as low as 21% for males and 25% for females. These findings show that most junior elite sprint swimmers were not able to maintain the same level of competitiveness in their senior careers. So far, the specific characteristics that underpin the successful development of swimmers who stay on track towards the senior elite level, as opposed to those who do not, remain unclear.

By following swimmers throughout the junior-to-senior transition and investigating underlying performance characteristics (e.g., anthropometrics, technical skills, muscle power, and maximal swimming velocity) in relation to their performance level at senior age, we may acquire a better understanding about the specific factors that contribute to progression toward elite level swimming performance at this challenging stage. Moreover, insight into the levels and development of underlying performance characteristics during the junior-to-senior transition extends the knowledge about general performance development towards expertise. This may not only enrich the field of sport science but also has the potential to enhance the efficacy and efficiency of athlete development programs by providing science-based reference for coaches and swimmers.

Therefore, the present study strived to gain a more profound understanding of the distinctions in development between swimmers who are considered to be on track to the senior elite level (referred to as high-performing seniors) compared to those who are not (referred to as lower-performing seniors) during the junior-to-senior transition (males aged 16–19 and females aged 15–18). We first examined whether high-performing seniors differed from lower-performing seniors in levels of swim performance and underlying characteristics when they were late juniors (males aged 17; females aged 16). Second, we investigated whether developmental differences in swim performance and underlying performance characteristics emerged during the junior-to-senior transition (males aged 16–19 and females aged 15–18) based on senior performance-level attainment. We hypothesized that high-performing seniors showed better and faster development on both swim performance and its underlying performance characteristics than lower-performing seniors during the junior-to-senior transition.

## 2 Materials and methods

### 2.1 Ethical approval

All participants were informed of the study’s procedures prior to their participation and provided their written informed consent to participate. Informed consent was also obtained from parents of participants who were below 16 years old. All procedures used in the study complied with the Helsinki Declaration and were approved by the research ethics committee of the University Medical Center Groningen, University of Groningen, Netherlands (202000488).

### 2.2 Participants

Participants were twenty-nine Dutch talented swimmers (12 males, 757 ± 110 World Aquatics Points; 17 females, 743 ± 82 World Aquatic Points) who progressed through the junior-to-senior transition (males aged 16–19; females aged 15–18). Swimmers were specialized in sprint (50–100 m; 8 males and 10 females) or middle-distance (200–400 m; 4 males and 7 females) events. According to the age group regulations of the Royal Dutch Swimming Federation (KNZB, 2023), swimmers were classified as late juniors (males aged 16–17; females aged 15–16) or early seniors (males aged 18–19; females aged 17–18) based on their calendar age on December 31st of the corresponding season (KNZB, 2022).

During their late junior years, all swimmers participated in one of the (initial) talent development (TD) programs of the KNZB, involving six to ten swim (in-water) training sessions per week. Additionally, they performed mobility training before every morning or afternoon swim session and took part in strength training, typically one to two times per week. Upon reaching early senior age, the group underwent further differentiation. Seventeen swimmers (9 males; 8 females) advanced to the subsequent, higher-level TD programs, while six (1 male and 5 females) swimmers remained in the initial TD program. Additionally, six (2 males and 4 males) swimmers were deselected from the program.

### 2.3 Study design

Longitudinal data on swim performance and underlying performance characteristics were collected over four swimming seasons. Performance data (season best times from all long course swim events) were obtained from Swimrankings (Swimrankings, 2022) at the end of each swimming season. Repeated measures of underlying performance characteristics were conducted as an integral part of all talent development programs, serving as the primary data source for this study.

**FIGURE 1:**
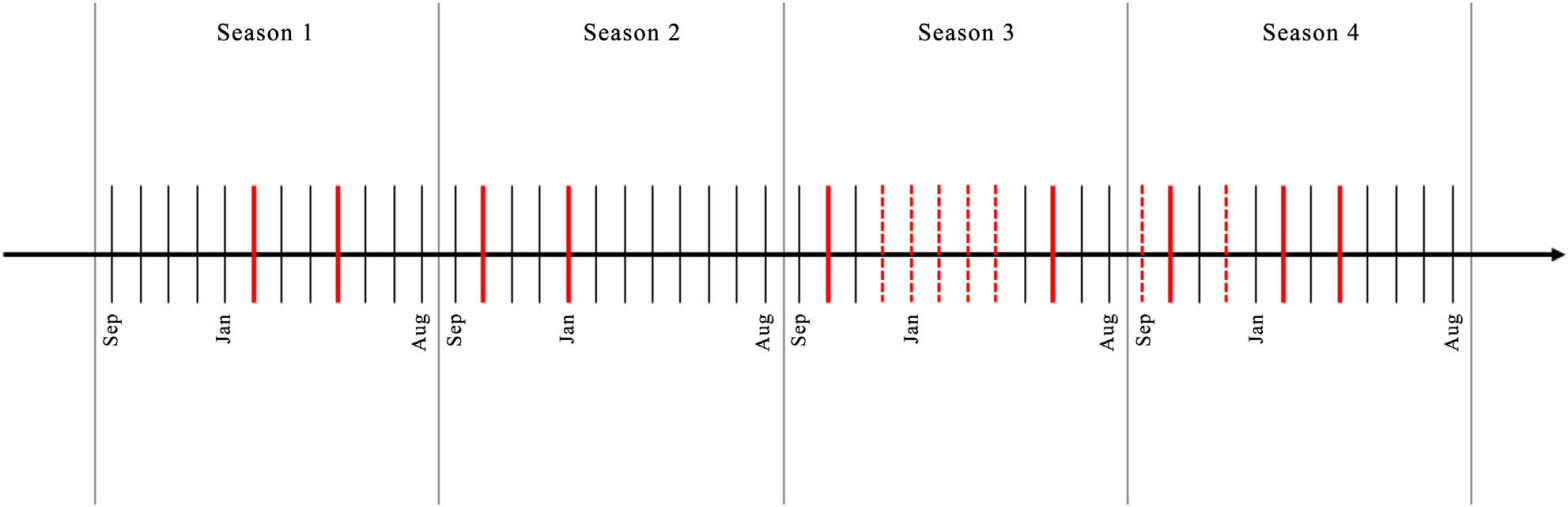
Timeline for data collection over four seasons. All measurement moments included assessment of height, CMJ, start, turn and sprint tests. The solid red line represents the measurement moments for the initial TD programs, while the dashed red line represents the measurement moments for the subsequent, higher-level TD programs.

The frequency of measurement moments varied depending on the specific TD program in which the swimmers were enrolled (see Figure 1). This ranged from two to three times a year for the initial TD programs (indicated by the solid red line) to once per month for the subsequent, higher-level TD programs (indicated by the dashed red line). According to coaches’ recommendations, the measurement moments were strategically scheduled to align with the competitive calendar for each specific season. The median number of observations was *n* = 6 in males and *n* = 10 in females.

### 2.4 Measurements

Each measurement moment consisted of land-based tests (height assessment and the countermovement jump test), followed by swimming tests. Additionally, swimmers provided their date of birth and reported their weekly training hours dedicated to strength, mobility and swim training using an online questionnaire (see Table 1).

**TABLE 1:** Descriptive characteristics of male and female swimmers at late junior age (17 and 16 years respectively) according to their performance level at early senior age.

	Males (N = 12)			Females (N = 17)		
	High-performing seniors (*n* = 6)	Lower-performing seniors (*n* = 6)	Effect sizes	High-performing seniors (*n* = 10)	Lower-performing seniors (*n* = 7)	Effect sizes
	M ± SD	M ± SD	d	M ± SD	M ± SD	d
Age (years)	17.6 ± 0.2	17.6 ± 0.2	0.0	16.5 ± 0.3	16.5 ± 0.3	0.0
Swim training (hours per week)	14.7 ± 2.7	16.7 ± 3.0	−0.7	16.0 ± 2.6	15.0 ± 1.1	0.5
Strength training (hours per week)	2.9 ± 1.5	2.2 ± 0.4	0.7	2.0 ± 0.5	2.8 ± 1.0	−1.1
Mobility training (hours per week)	1.7 ± 0.5	1.9 ± 0.4	−0.4	2.3 ± 0.8	2.0 ± 0.8	0.2
Height (cm)	188.1 ± 8.0	185.7 ± 5.7	0.3	177.4 ± 7.2	172.4 ± 3.9	0.8
rLBP (Watt/kg)	41.0 ± 5.7	35.8 ± 10.0	0.6	32.8 ± 3.0 *	28.0 ± 4.8	1.2
rStart (%)	106.1 ± 5.3 *	114.3 ± 5.2	−1.6	109.9 ± 5.0	110.3 ± 6.8	0.0
rTurn (%)	98.3 ± 3.1 **	106.1 ± 2.9	−2.6	100.0 ± 4.3	103.4 ± 3.6	−0.8
rSprint (%)	100.8 ± 2.6 ***	93.0 ± 2.0	3.4	100.8 ± 3.6 *	95.1 ± 3.0	1.7
rSI (%)	82.8 ± 11.5	83.0 ± 7.5	0.0	95.1 ± 6.0 *	79.2 ± 10.0	2.0
rST at late junior age (%)	107.4 ± 2.4 **	114.7 ± 2.9	−2.7	109.1 ± 1.4 *	115.0 ± 3.7	−2.3
rST at early senior age (%)	106.1 ± 1.5 **	111.8 ± 2.0	−3.3	107.9 ± 1.5 *	114.7 ± 4.2	−2.4

Note. 13 freestyle (6 males); 5 butterfly (1 male); 6 breaststroke (3 males); 5 backstroke (2 males) swimmers. Age refers to the calendar age as of December 31st of the corresponding season. rLBP, relative lower body power; rStart, relative start time; rTurn, relative turn time; rSprint, relative maximal swimming velocity; rSI, relative stroke index; rST, relative swim time. *p < 0.05 (one-tailed); **p < 0.01 (one-tailed); ***p < 0.001 (one-tailed).

#### 2.4.1 Height

Swimmers’ height was assessed using a stadiometer (Seca, 217, Seca GmbH & Co.KG, Germany), which provided a measurement accuracy of 0.1 cm. Measures were taken twice and conducted by the same two researchers. The mean value was documented. A third measure was taken if the difference between the first two exceeded 0.4 cm. The median was then recorded.

#### 2.4.2 Countermovement jump test

Swimmers were instructed to perform three double-leg vertical countermovement jumps (CMJ) without arm swing, which is reported as a valid and reliable test to measure lower body power (Markovic et al., 2004). Lower body power is considered to be of particular importance during starts and turns, as it is in these moments that the lower extremities must generate the greatest impulse to achieve the highest accelerations off the block and wall respectively (West et al., 2011; Jones et al., 2018; Keiner et al., 2021). The jumps began from an upright position, and there was a short break (∼2 s) between each trial to allow the swimmers to return to the starting position. Each trial was recorded with a linear position transducer (GymAware PowerTool, GymAware, Australia), which has shown to be a reliable and valid instrument for profiling various variables, including mean power (Cronin et al., 2004). The PowerTool was placed next to the swimmer, clear of their feet. To create an attachment point for the tether, swimmers held a broomstick across their shoulders with their hands. Relative lower body power (rLBP), calculated as the average (concentric) mean power (Watt) over three jumps divided by the swimmer’s weight (kilograms), was taken as outcome measure for further analyses.

#### 2.4.3 Swimming tests

Swimming tests consisted of starts, turns and sprints, which swimmers performed in their best stroke with maximal effort, while wearing racing suits. Each test was conducted twice before proceeding to the next. Between efforts, swimmers rested for three till 5 minutes according to coaches’ advice.

Swimming tests were recorded with four underwater digital video camera’s (50 Hz, Basler scout, scA1400-30gc, Basler, Germany), positioned on the lateral side of the pool at the 2.5-, 5-, 10-, and 15-m marks, respectively. In addition, the block and flight phase of the start were recorded with a digital video camera above the water, perpendicular to the starting block. Kinematic data of starts, turns and sprints were extracted from these recordings using time video analysis.

##### 2.4.3.1 Starts

Swimmers were instructed to perform a complete start, including a still position on the block or backstroke ledge, the underwater phase, breakout and subsequent swimming phase. Swimmers were directed to keep swimming until their head reached a distance of 17-m, which was indicated with a marker at the bottom of the pool. This marker ensured that they successfully surpassed the 15-m mark with maximum velocity. Starts were performed with the same material requirements as in major international swimming competitions (starting block with equal dimensions as the Omega OSB11 or the Omega OBL2 PRO backstroke ledge). Start time, defined as time between starting signal (light trigger of the starting device visible in the video footage) until the head of the swimmer passed the 15-m mark, was taken as outcome measure for further analyses (Morais et al., 2019; Born et al., 2022b).

##### 2.4.3.2 Turns

Swimmers were instructed to perform a complete turn including the underwater phase, break-out and subsequent swimming phase. Swimmers were directed to start their effort at 12.5-m before the wall and to end their effort when their head reached a distance of 17-m, which was indicated with a marker at the bottom of the pool. This marker ensured that they successfully surpassed the 15-m mark with maximum velocity. Turns were performed with the same material requirements as in major international swimming competitions (Omega touch pad on the wall). Turn time, defined as time between 5-m in (head of the swimmer at the 5-m mark before the wall) and 15-m out (head of the swimmer at the 15-mark out of the wall), was taken as outcome measure for further analyses (Morais et al., 2019; Born et al., 2022b).

##### 2.4.3.3 Mid-pool sprints

Swimmers were instructed to perform a 25-m distance sprint at maximal swimming velocity, starting in the middle of a 50-m pool. Their effort was completed when they touched the wall. Maximal swimming velocity was defined as the clean swimming velocity (10-m distance divided by time for the 10-m distance) between the 10- and 20-m segment of the 25-m trial. Stroke rate (cycles·min^−1^) was calculated as the number of strokes completed by the swimmer during this 10-m segment (Poujade et al., 2002), one stroke rate cycle being defined as the time between the entry of one hand until the following entry of the same hand (Huot-Marchand et al., 2005). Stroke length (m·cycle-1) was calculated as the ratio between swimming velocity over the 10-m segment and the corresponding stroke rate (Poujade et al., 2002). Stroke index (SI), an indirect measure of swimming efficiency, was calculated by multiplying swimming velocity by stroke length. The SI measures the ability of the swimmer to complete a given distance with a particular speed in the fewest possible number of strokes (m^2^·s^−1^·cycle-1) (Costill et al., 1985). Maximal swimming velocity and SI were taken as outcome measures for further analyses.

### 2.5 Data processing

To enable comparisons among swimmers specialized in different strokes and distances, outcomes were related to meaningful reference values and expressed as a percentage, rather than absolute values (see Eq. 1). Specifically, swim time was related to the prevailing world record (WR), a method initially introduced by Stoter et al. (2019) in speed skating and subsequently applied in competitive swimming (Post et al., 2020a; Post et al., 2020b). Lower percentages on relative Swim Time (rST) indicated swim performances closer to the WR.

Furthermore, scores on swimming tests were related to the average start time, turn time, clean swimming velocity and SI of male and female finalists at the European Championships in 2021 (Born et al., 2022b). Stroke-specific data of the 100- and 200-m events were used as reference values for sprinters (50–100-m) and middle-distance (200–400-m) swimmers in our sample respectively (see Supplementary Appendix SB). Higher percentages on relative maximal swimming velocity (rSprint) and stroke index (rSI), and lower percentages on relative start- (rStart) and turn time (rTurn), indicate scores more close to the European elite level (set to 100%). For example, the 15-m start time of a junior male freestyle sprinter (6.20 s) was related to the average 15-m start time of the 100-m freestyle European male finalists (5.55 s), resulting in a relative start time of 111.7% [(6.20/5.55) * 100%].

$$relative\;variable\;x=\frac{absolute\;variable\;x}{reference\;value\;x}\times 100\%$$ (1)

### 2.6 Data selection

Rather than considering all observations, we selected the swimmers’ season best rST, rStart, rTurn, rSprint along with the corresponding rSI, and rLBP for further analyses (see Supplementary Appendix SA for number of measurements by performance level group and age category). Any other data were excluded, minimizing the impact of variations in achievements within a season. The median number of between-season observations was *n* = 3 in males and females.

### 2.7 Defining performance level groups

A higher- and lower-level performance group were defined according to performance trajectories of international elite swimmers, representing a performance level similar to the top 50 swimmers worldwide of the past 5 years (2017–2022 with the exception of 2020, see Post et al., 2020a). Following the approach adopted in previous studies (Stoter et al., 2019; Post et al., 2020b), the maximum season best rST by age category, sex and swim event of these international elite swimmers was used as performance benchmark (%WR, see Supplementary Appendix SC). Swimmers whose season best rST at early senior age (males aged 18–19; females aged 17–18) fell within the corresponding performance benchmark were categorized as high-performing seniors and considered to be on track to reach the elite level (6 males; 10 females). Conversely, swimmers who did not meet the performance benchmark were classified as lower-performing seniors and considered to be off track to reach the elite level (6 males; 7 females). To illustrate, consider a 19-year-old male swimmer competing in the 100 m freestyle. If his season best rST is 107.9%, he would be classified in the high-level performance group since it falls within the performance benchmark for 19-year-old males in the 100 m freestyle, which is set at 108.9%. However, if his season best rST is 110.0%, he would be classified in the lower-level performance group as it exceeds the corresponding performance benchmark.

### 2.8 Statistics

All data were analyzed for males and females separately, using R (R Core Team, 2021). Data were initially screened on outliers (using box plots), normality (using QQ-plots) and homogeneity of variance (using Levene’s test). Outliers (5 in males; 5 in females) were acknowledged as a natural occurrence within the population and, consequently, were not removed from the dataset. Normality was violated in males (strength training, height, rLBP, rStart, and rST at early senior age) and females (swim-, strength-, and mobility training, height and rSI). Homogeneity of variance was assumed with the exception of rST at late and early junior age in females.

Cross-tabulation analyses were performed to analyze the relationship between performance level group at early senior (males aged 18-19; females aged 17-18) and late junior age (males aged 17; females aged 16). For high- and lower-performing seniors, mean scores and standard deviations were calculated for swim performance and underlying performance characteristics at the beginning of their junior-to-senior transition (males aged 17; females aged 16). Independent sample t-tests were included to examine between-group differences on age, swim-, strength-, and mobility training (hours per week), height, rLBP, rStart, rTurn, rSprint, rSI, rST at late junior age and rST at early senior age (to ensure correct definition of our performance groups). Mann-Whitney U tests were included to examine between-group differences on variables in which assumptions were violated. For all tests, *p* < 0.05 (one-tailed) was considered statistically significant.

To interpret the scores, effect sizes (Cohen’s d values) were calculated. An effect size of approximately 0.20 was considered small, while effect sizes of 0.50, 0.80 and 1.20 were considered medium, large and very large, respectively (Cohen, 1988). A sensitivity power analysis confirmed that our statistical tests were sufficiently sensitive to detect significant differences between performance level groups with a minimum detectable effect size of 1.5 and 1.3 (males and females respectively) (alpha = 0.05, power = 0.80). Statistical tests for measuring invariance were not performed given the nature of our dataset (relatively few observations for many items).

Longitudinal multilevel models were created to describe development of rST, rStart, rTurn, rSprint, rSI, and rLBP (dependent variables) as a function of (chronological) age, using the lmer4 package in R (R version 3.6.0). The age effect (which was used as measure for development over time) was not imposed to be identical between high- and lower-performing seniors. Therefore, a nested interaction between age and performance level group at early senior age was included. To represent these two performance level groups in the statistical models, one dummy variable (high-level performance group) was included and the lower-level performance group functioned as reference level. A random intercept model was selected as the most appropriate variance structure, allowing the inclusion of each swimmer’s individual trajectory that randomly deviates from the average population trajectory. In sum, the following multilevel model was adopted:

$$Y_{is}=\alpha_{i}+\beta_{1}\times {Age}_{is}+\beta_{2}\times {Age}_{is}\times {High-level\;performance\;group}_{i}+u_{i}+\varepsilon_{is}$$ (2)

$$u_{i}\;∼\;N\;\left(0,\sigma_{0}^{2}\right)$$

$$\varepsilon_{is}\;∼\;N\left(0,\sigma^{2}\right)$$

$Y_{is}$ was the dependent variable (e.g., rSprint) for swimming season s of swimmer i , $\alpha_{i}$ the intercept of swimmer i , ${Age}_{is}$ the corresponding age value and ${High-level\;performance\;group}_{i}$ the dummy variable indicating whether or not swimmer i was in the high-level performance group. The unexplained information was the sum of $u_{i}$ (between-subject variance) and $\varepsilon_{is}$ (residual variance). The models were validated by using visible patterns in residual plots to check violations of homogeneity, normality and independence. Predictor variables were considered significant if the *p*-value of the estimated mean coefficient is smaller than 0.05.

## 3 Results

Table 1 shows the descriptive statistics, including effect sizes, of male and female swimmers at late junior age (males aged 17; females aged 16) according to their performance level at early senior age. High-performing senior swimmers outscored lower-performing seniors on rST at early senior age (*p* < 0.05; very large effect sizes), confirming a correct definition of performance level groups in both males and females. No significant differences between groups on age and weekly swim-, strength-, and mobility training hours were found (*p* > 0.05).

High-performing senior males scored significantly higher on rSprint (*p* < 0.001), and lower on rStart (*p* < 0.05), rTurn (*p* < 0.001) and rST (*p* < 0.01) at age 17 compared to lower-performing peers. The effect sizes in these four variables were very large. Although not statistically significant, high-performing senior males had higher scores on height (small to medium effect sizes) and rLBP (medium to large effect sizes) at age 17 compared to lower-performing males. Similar scores between groups were found on rSI (no effect).

High-performing senior females scored significantly higher on rLBP (*p* < 0.05), rSprint and rSI (*p* < 0.05), and lower on rST (*p* < 0.05) at age 16 compared to lower-performing peers. The effect sizes in these four variables were very large. Although not statistically significant, high-performing senior females had higher scores on height (medium to large effect sizes) and lower scores on rTurn (large effect sizes) at age 16 compared to lower-performing peers. Similar scores between groups were found on rStart (no effect).

Table 2 shows the cross-tabulation analyses of the relationship between performance level group at early senior and late junior age of male and female swimmers. At early senior age (18–19 years), six of the twelve male swimmers (50%) were classified in the high-level performance group. All six high-performing male seniors (100%) were also categorized as high-performing juniors (16–17 years), whereas four out of the ten (40%) high-performing male juniors switched to the lower-level performance group at early senior age. For females, ten of the seventeen swimmers (59%) were classified in the high-level performance group at early senior age (17–18 years). All ten high-performing female seniors (100%) were also categorized as high-performing juniors (15–16 years), whereas three out of the thirteen high-performing junior females (23%) switched to the lower-level performance group at early senior age.

**TABLE 2:** Cross-tabulation analyses of the relationship between performance level group at early senior and late junior age of male and female swimmers.

	Total (N)	High-performing juniors (n)	Lower-performing juniors (n)
*Males*	12 (100%)	10 (83%)	2 (17%)
High-performing seniors	6 (50%)	6 (100%)	0 (0%)
Lower-performing seniors	6 (50%)	4 (67%)	2 (33%)
*Females*	17 (100%)	13 (76%)	4 (24%)
High-performing seniors	10 (59%)	10 (100%)	0 (0%)
Lower-performing seniors	7 (41%)	3 (43%)	4 (57%)

Note. Swimmers whose relative season best performaces at late junior age (males 16–17 years, females 15–16 years) fell within the performance benchmark were categorized as high-performing juniors. Conversely, those swimmers who were not fast enough were classified as lower-performing juniors.

### 3.1 Developmental models according to performance level group at early senior age

Table 3 shows the developmental models on rST, rStart, rTurn, rSprint, rSI, and rLBP created for males and females. Each model consists of two age effects, which allows for different rates of development between high- and lower-performing seniors. The “age” term denotes the development of lower-performing seniors, whereas “age + age × high-level performance group” denotes the development of high-performing seniors. To illustrate (using the fixed effects of the model only), the rST for a high-performing senior male at age 17 will be predicted as follows:

$$rST=128.09+\left(-0.79\times 17\right)+\left(-0.34\times 17\right)=108.88$$ (3)

**TABLE 3:** Model estimates for male (N = 10 with 28 observations) and female (N = 14 with 39 observations) swimmers.

*Males*	rST		rStart		rTurn		rSprint		rSI		rLBP	
	*Estimates (S.E.)*	*p-value*	*Estimates (S.E.)*	*p-value*	*Estimates (S.E.)*	*p-value*	*Estimates (S.E.)*	*p-value*	*Estimates (S.E.)*	*p-value*	*Estimates (S.E.)*	*p-value*
*Fixed effects*												
Intercept	128.09 (4.41)	**<0.001**	142.88 (7.59)	**<0.001**	118.59 (8.94)	**<0.001**	94.76 (5.81)	**<0.001**	73.08 (17.49)	**0.001**	−9.57 (22.66)	0.340
Age	−0.79 (0.24)	**0.002**	−1.70 (0.42)	**<0.001**	−0.72 (0.49)	0.082	−0.12 (0.32)	0.359	0.71 (0.96)	0.236	2.62 (1.26)	**0.030**
Age × high-level performance group	−0.34 (0.09)	**<0.001**	−0.31 (0.18)	0.051	−0.35 (0.11)	**0.002**	0.45 (0.08)	**<0.001**	−0.13 (0.25)	0.304	0.19 (0.24)	0.222
*Random effects*												
σ^2^	0.85		2.64		4.46		1.79		16.11		18.29	
τ_00_	6.48		25.97		7.74		4.26		45.54		40.24	
Marginal *R* ^2^/Conditional *R* ^2^	0.61/0.95		0.31/0.94		0.50/0.82		0.74/0.92		0.02/0.74		0.16/0.74	

***Females***	rST		rStart		rTurn		rSprint		rSI		rLBP	
	*Estimates (S.E.)*	*p-value*	*Estimates (S.E.)*	*p-value*	*Estimates (S.E.)*	*p-value*	*Estimates (S.E.)*	*p-value*	*Estimates (S.E.)*	*p-value*	*Estimates (S.E.)*	*p-value*
*Fixed effects*												
Intercept	112.95 (3.34)	**<0.001**	135.38 (6.14)	**<0.001**	116.76 (6.66)	**<0.001**	97.00 (8.22)	**<0.001**	81.94 (12.61)	**<0.001**	−6.40 (13.12)	0.316
Age	−0.02 (0.20)	0.454	−1.78 (0.38)	**<0.001**	−0.97 (0.41)	**0.012**	0.03 (0.49)	0.479	−0.06 (0.76)	0.467	2.44 (0.78)	**0.003**
Age × high-level performance group	−0.21 (0.06)	**0.001**	0.26 (0.17)	0.075	0.01 (0.15)	0.482	0.20 (0.12)	0.059	0.76 (0.23)	**0.002**	−0.22 (0.16)	0.089
*Random effects*												
σ^2^	0.87		2.77		3.59		6.36		14.32		11.89	
τ_00_	2.71		24.25		16.10		10.01		40.19		14.86	
Marginal *R* ^2^/Conditional *R* ^2^	0.43/0.86		0.15/0.91		0.05/0.83		0.13/0.66		0.38/0.84		0.22/0.65	

Note. “Age” term denotes development of lower-performing seniors. “Age + Age × high-level performance group” denotes development of high-performing seniors. Abbreviations: rST (relative swim time); rStart (relative start time); rTurn (relative turn time); rSprint (relative maximal sprint velocity); rSI (relative stroke index); rLBP (relative lower body power). Significant predictor variables (*p* < 0.05) are denoted in bold with adjustments made for one-sided hypothesis testing in the calculation of the *p*-value.

Given the study’s primary focus on differences between high- and lower-performing swimmers, particular emphasis will be placed on analyzing the interaction term (age × high-level performance group). A significant interaction term would indicate a faster rate of development of high-performing swimmers compared to their lower-performing peers.

**FIGURE 2:**
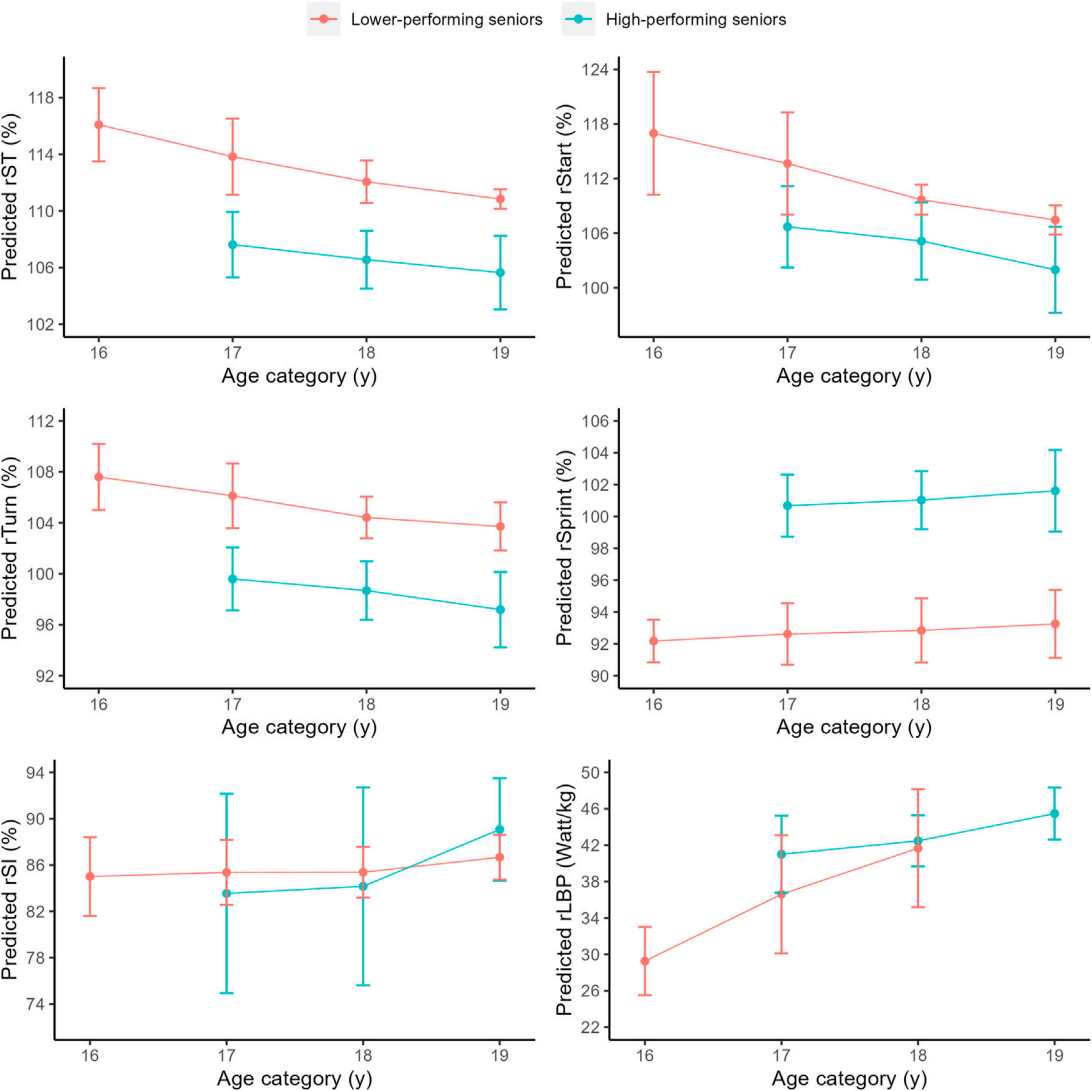
Predicted development as function of age (mean ± SD) of swim performance and underlying performance characteristics in males (N = 10 with 28 observations).

**FIGURE 3:**
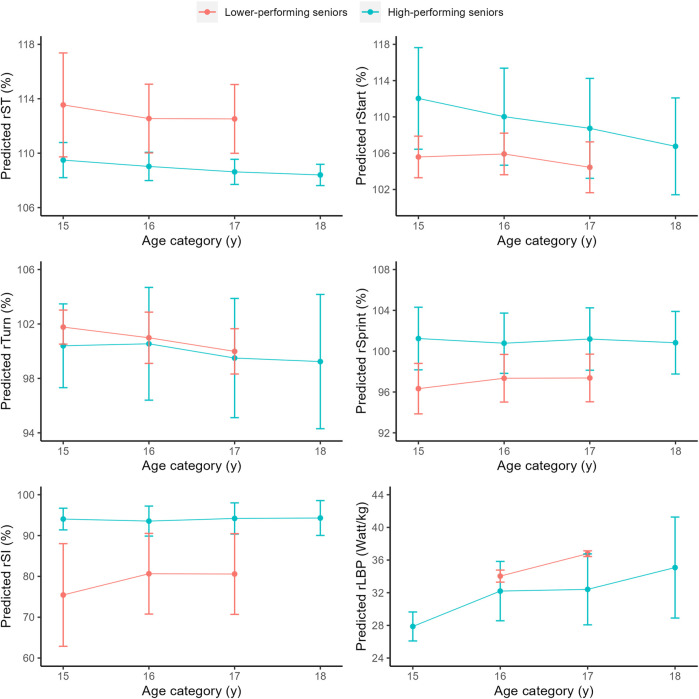
Predicted development as function of age (mean ± SD) of swim performance and underlying performance characteristics in females (N = 14 with 39 observations).

In males, high-performing senior swimmers showed significant faster progression over time on rST (*p* < 0.001), rTurn (*p* < 0.01) and rSprint (*p* < 0.001) compared to lower-performing senior swimmers. In females, high-performing senior swimmers showed significant faster progression over time on rST (*p* < 0.01) and rSI (*p* < 0.01). No significant developmental differences between groups were found on rStart and rLBP (males and females), rSI (males only) and rTurn and rSprint (females) (*p* > 0.05). Figure 2 (males) and Figure 3 (females) reflect the predicted development of high- and lower-performing seniors during the junior-to-senior transition.

## 4 Discussion

The present study strived to gain a more profound understanding of the distinctions in development between swimmers who are considered to be on track to the senior elite level (referred to as high-performing seniors; 6 males and 10 females) compared to those who are not (referred to as lower-performing seniors; 6 males and 7 females). Retrospectively studying these swimmers, we found that high-performing seniors (males aged 18–19 and females aged 17–18) outperformed their lower-performing peers on most of the assessed underlying characteristics at late junior age (males aged 17; females aged 16). Furthermore, high-performing seniors were characterized with significantly faster development in season best performances (for both males and females), maximal swimming velocity and turns (males), and SI (females) during the junior-to-senior transition (males aged 16–19; females aged 15–18).

### 4.1 Performance

Our findings showed that high-performing seniors were already on track to the elite level at age 17 (males) and age 16 (females). At this particular age, it became evident that these swimmers demonstrated significantly faster season best performances compared to their lower-performing peers (very large effect sizes), which aligns with previous research of Post et al. (2020a). Moreover, we found that high-performing seniors showed significantly faster development of swim performance during the junior-to-senior transition. This further amplified their initial advantages over lower-performing peers.

While all high-performing seniors were classified as high-performing juniors, it is important to note that none of the lower-performing juniors transitioned to the high-performing senior group. This observation indicates that bridging the performance level gap faced by lower-performing juniors is exceptionally challenging. Moreover, it suggests that the development of swim performance becomes more stable during the late junior years (males aged 16–17; females aged 15–16), which is in line with previous work of Costa et al. (2011). As such, we state that the importance of performance level increases as swimmers approach their age of peak performance (Allen and Hopkins, 2015), and that a high level of swim performance at late junior age (i.e., being on track), may be required to advance to the senior elite level, which was also observed in other individual sports like cycling (Gallo et al., 2022; Mostaert et al., 2022). Additionally, we have found that not all high-performing juniors ended up as high-performing seniors. This observation highlights that being on track at late junior age does not guarantee the successful continuation to the senior elite level, which aligns with previous studies (Barreiros et al., 2014; Brustio et al., 2021). Furthermore, it underscores the difficulty of sustaining an upward trajectory towards swimming expertise, thereby counteracting the commonly observed plateau in progress that tends to occur during the junior-to-senior transition (Born et al., 2022a).

### 4.2 Underlying performance characteristics and its development

A closer analysis of the assessed underlying characteristics of swim performance revealed that high-performing seniors were taller (small effect sizes in males; large effect sizes in females) and significantly outperformed lower-performing peers in terms of maximal swimming velocity (very large effect sizes) at late junior age (males aged 17; females aged 16). These findings align with previous studies that have reported advantageous anthropometrics and higher swimming speed among faster swimmers, particularly in the youth category (Morais et al., 2017; Barbosa et al., 2019; Morais et al., 2022). It is noteworthy that both male and female high-performing seniors exhibit swimming speeds at late junior age that are nearly comparable to those of finalists of the European Championships in 2021, as evidenced by the values approaching 100%. Additionally, high-performing senior females demonstrated significantly higher SI at late junior age compared to their lower-performing peers (very large effect sizes). This finding corresponds with existing literature showing that faster (early junior) swimmers distinguished themselves from others with better SI (Barbosa et al., 2019; Morais et al., 2021). However, contrary to our initial hypothesis, no differences in SI were observed among males. Given that their SI scores are the farthest from reaching values close to 100%, overall swimming efficiency seems to be the (relatively) weakest point for males when compared to other variables at late junior age.

It is important to note that both maximal swimming velocity and SI were derived from the 25-meter sprint test, and therefore, they should be considered together. When considering these variables collectively, it can be concluded that high-performing seniors demonstrated higher maximal swimming velocity with the same (males) or even higher levels of SI (females) at late junior age compared to their peers. This may be an important advantage as swimmers need to maintain optimal power output in an efficient and skillful manner throughout the event (Miyashita, 1996). Moreover, we found that high-performing seniors demonstrated significantly faster rates of progression on maximal swimming velocity (males) and SI (females) during the junior-to-senior transition. Notably, it is precisely in these variables that lower-performing peers experienced a plateau in their progress (as evidenced by beta values close to zero), indicating that high-performing seniors were extending their advantages even further over time.

As expected, high-performing seniors demonstrated higher lower body power (medium effect sizes in males; very large effect sizes and significant in females) and faster turns (very large effect size and significant in males; large effect sizes in females) compared to lower-performing seniors at late junior age. In the case of turns, high-performing males demonstrated significantly faster rates of progression. Moreover, starts were significantly faster for high-performing senior males (very large effect sizes) at late junior age, whereas no differences were observed among females. When compared to other variables, it is evident that females’ starts are their relatively weakest point, as their start performances show the greatest deviation from values close to 100%. It is worth noting, however, that our study assessed the total start and turn times and did not explore specifically the various components involved, such as the block/push-off phase, underwater phase or clean swimming phase. By conducting more detailed investigations of these components in future studies, we could attain a more comprehensive understanding of the specific phases in which differences in starts and turns emerge. Moreover, the inclusion of measures related hydrodynamics, power output in the water and aerobic capacity could offer insights into the mechanisms behind the observed distinctions between high- and lower-performing swimmers in the present study.

### 4.3 Training

Our findings are inherently connected to both the quantity and quality of swim training. As such, inter-individual variations in training characteristics could help explain our results. Our study suggested that high-performing senior females tend to be involved in more weekly swim training hours compared to their lower-performing peers at age 16 (medium to large effect sizes). This suggests that high-performing females spent more time in the water to work on their skills, which may have benefitted their progression (Baker and Young, 2014). It is important to note, however, that the increase in swim training hours does not automatically translate to higher performance levels, which is evidenced by high-performing senior males who appear to have participated in fewer weekly swim training hours compared to their lower-performing peers at age 17 (medium to large effect sizes). This could indicate that high-performing males derived more from their training sessions in terms of quality.

The quality of training encompasses factors such as self-regulation of learning (SRL). SRL indicates the extend to which individuals are metacognitively, motivationally and behaviorally proactive in their own learning processes (Zimmerman, 1986; 2006). Previous research on SRL in swimming showed that youth swimmers on track to the elite level are characterized by more frequent use of reflection processes during training and evaluation processes after training, which suggest that they learn and train in a more efficient and effective manner (Post et al., 2022). Ultimately, this could contribute to a higher quality of daily training, which may result in greater improvements during a season and higher performance levels. As talented swimmers approach the senior elite level, the difficulty of making progress increases significantly (Born et al., 2022a), partly due to the principle of diminishing returns of training (Hoffman, 2014). Hence, SRL processes may become of particular importance during the junior-to-senior transition.

Moreover, a swimmer’s coach plays an essential role in the quality of training. Depending on the coach’s vision of swimmers’ performance development, specific aspects of swimming performance (such as starts, turns, or technique) are emphasized in the training program (Marinho et al., 2020). Combined with a swimmer’s training history, fitness level, and specialization, a personalized training approach is designed, including strength-, and mobility training. Therefore, future studies investigating the inter-individual differences in these training characteristics and their relation to the development of swim performance and underlying factors would be of great value in advancing our understanding of the pathway to swimming expertise.

### 4.4 Strengths and weaknesses

The uniqueness of the present study lies in its integration of study design, sample, and analysis, which sets it apart from other studies in multiple ways. First of all, our longitudinal analysis of performance and multiple underlying performance characteristics (multi-dimensional approach) allows for a more comprehensive understanding of the complex nature of athlete development, resulting in a more nuanced and insightful analysis of progression towards elite level swimming performances. Second and unlike previous studies, we followed top-tier national age group swimmers during their junior-to-senior transition. We particularly focused on the late junior and the early senior years, a time span of 4 years at the end of the talent trajectory (males aged 16–19; females aged 15–18). It is worth noting that this specific group of swimmers has been underrepresented in existing research, with an even greater lack of focus on female athletes. As such, the present study shed a light on the unique developmental characteristics of talented male and female swimmers, revealing both similarities and differences between sexes. This underscores the importance of recognizing that findings from male swimmers cannot be directly extrapolated to females, emphasizing the need for sex-specific considerations and individualized approaches. Third, our analysis focused on differences between high-performing and lower-performing senior swimmers in relation to international reference values, while considering different rates of development between these performance level groups in our models. Opposite to their lower-performing peers, high-performing seniors are considered to be on track to the senior elite level. This group division was defined by benchmarks derived from the observed developmental pathway of international elite swimmers who ranked among the top 50 worldwide. Moreover, scores on swimming tests were related to (in-competition) levels of starts, turns, maximal swimming velocity and SI achieved by finalists of the European Championships in 2021. This comparison enabled us to assess the level of late-junior swimmers in relation to the level they need to attain as senior elite swimmers. Taken together, the present study is the first to provide evidence for differences in developmental pathways (both on performance and its underlying characteristics) of both male and female senior swimmers who are on track to the elite level compared to those who are not during the junior-to-senior transition.

Alongside the strengths, it is important to acknowledge and address the limitations that exist within the present study. Unsurprisingly, we faced the challenge of a relatively small sample sizes, which is inherent in elite sports research (Skorski and Heckseden, 2021). As a result, the statistical power of our analysis was constrained, limiting our ability to detect anything other than substantial differences between groups. This limitation increases the likelihood of interpreting minor changes in variables as having no effect, emphasizing the need for cautious interpretation of the study’s findings. However, it is essential to recognize that even subtle changes can hold practical significance, particularly in the context of elite sports (Gabbett et al., 2017). Therefore, to ensure a comprehensive interpretation of our results, we placed particular emphasis on effect sizes. Effect sizes provide a measure of the magnitude of the observed effects (Nuzzo, 2014), allowing us to evaluate the practical significance of even the smallest changes. Additionally, we implemented a data pooling strategy to increase our sample size by combining the data from all our swimmers. However, due to this approach, we were unable to include stroke-specific analyses and stroke-specific variables, such as stroke rate, in the present study.

Furthermore, it is worth emphasizing that not all swimmers in our study sustained their involvement in TD programs throughout the junior-to-senior transition. Therefore, we cannot rule out a survivorship bias given that our measurements of underlying performance characteristics were exclusively conducted among swimmers who remained in these programs. Consequently, the outcomes of our study specifically pertain to swimmers who remained in the system, reflecting the coach’s belief that a swimmer has the potential to make it to the senior elite level. It is recommended that future studies attempt to account for all swimmers initially involved in these kinds of measurements; however, this is challenging as swimmers who are deselected from talent development programs may not continue their efforts in the same way or may choose to pursue alternative career paths and retire (known as self-selection; Biele et al., 2019). Finally, the COVID-19 pandemic occurred during the study period and may have introduced potential confounding factors, such as periods of detraining, which could have influenced our findings (Zacca et al., 2019; Ruiz-Navarro et al., 2022). These factors must be considered when interpreting the findings of our study and in applying them to broader contexts of talent development in swimming (Elferink-Gemser and Visscher, 2012).

### 4.5 Perspective

The present study advances our understanding of progression towards elite level swimming performance in sprint and middle-distance events. Specifically, it underscores the significance of high initial levels of swim performance and underlying characteristics at late junior age (within 10% of international elite reference values, except for SI in males) as well as the ability to keep progressing on season best performances, maximal swimming velocity and turns (males) and SI (females) during the junior-to-senior transition. These may be crucial factors in the attainment of swimming expertise. Coaches and swimmers could focus on developing these underlying characteristics while being mindful of the differences in developmental profiles between males and females and tailor their training programs accordingly. Moreover, the study’s insights into the scores and developmental patterns of high-performing seniors could support coaches in monitoring their swimmers’ progression towards the elite level. However, coaches should consider these findings as a starting point rather than an endpoint for further development, as performance levels are influenced by unique combinations of underlying characteristics in which relative weaknesses can be compensated with strengths. Furthermore, it is important for coaches to be aware that for swimmers who are close to achieving 100% scores on swimming tests, ongoing development is crucial. This development is necessary to effectively bridge the gap between performance in isolated tests and performance in actual competitions. As our findings show that differences between high- and lower-performing seniors manifest at least at late junior age (males aged 17; females aged 16), it would be interesting to further investigate the earlier stages of their junior years. This could help elucidate when these differences first emerge as well as the factors that facilitate or hinder swimmers’ performance and progression, such as biological and environmental variables (e.g., maturation, training and selection procedures).

## Data Availability

The datasets presented in this article are not readily available because access permissions are with the principal investigator of the research project. Requests to access the datasets should be directed to m.t.elferink-gemser@umcg.nl.
