# Supplementary material for: Tracking performance and its underlying characteristics in talented swimmers: a longitudinal study during the junior-to-senior transition
Source: Front Physiol. 2023 Aug 9;14:1221567. doi: 10.3389/fphys.2023.1221567 (PMC10446966; doi:10.3389/fphys.2023.1221567)
Supplement: Supplementary file 1 [file Table1.docx]

Supplementary Material

Tracking performance and its underlying characteristics in talented swimmers: A longitudinal study during the junior-to-senior transition

Aylin K. Post^*^, Ruud H. Koning, Chris Visscher, Marije T. Elferink-Gemser

*** Correspondence:** Aylin Post: a.k.post@umcg.nl

# Supplementary Figures and Tables

Appendix A.

| Appendix A. Number of swimmers measured in one through four seasons and number of season best observations per age category during the junior-to-senior transition. | | | | | | | | | | | |
| --- | --- | --- | --- | --- | --- | --- | --- | --- | --- | --- | --- |
|  | N swimmers | n swimmers  measured for 1 season | n swimmers measured for  2 seasons | n swimmers measured for  3 seasons | n swimmers measured for  4 seasons | N  obs. | n obs. (at age 15) | n obs. (at age 16) | n obs. (at age 17) | n obs. (at age 18) | n obs. (at age 19) |
| *Males* |  |  |  |  |  |  |  |  |  |  |  |
| High-performing seniors | 6 | 1 | 2 | 3 | 0 | 14 | - | 0 | 6 | 5 | 3 |
| Lower-performing seniors | 6 | 1 | 1 | 3 | 1 | 16 | - | 3 | 6 | 4 | 3 |
|  |  |  |  |  |  |  |  |  |  |  |  |
| *Females* |  |  |  |  |  |  |  |  |  |  |  |
| High-performing seniors | 10 | 0 | 1 | 9 | 0 | 29 | 4 | 10 | 9 | 6 | - |
| Lower-performing seniors | 7 | 3 | 2 | 2 | 0 | 13 | 2 | 7 | 4 | 0 | - |
|  |  |  |  |  |  |  |  |  |  |  |  |
| *Total* | 29 | 5 | 6 | 17 | 1 | 72 | 6 | 20 | 25 | 15 | 6 |
| Note. N swimmers = total number of unique swimmers participating in the study; n swimmers = number of swimmers measured in one through four seasons; N obs. = total number of season best observations; n obs. = number of season best observations per age category. | | | | | | | | | | | |

Appendix B.

| Appendix B. References values of key performance indicators of European male and female finalists (retrieved from Born et al 2022). | | | | |
| --- | --- | --- | --- | --- |
|  | Clean swimming speed (m/s) | Stroke index | Start time (s) | Turn time (s) |
| *Males (100-m events)* |  |  |  |  |
| Backstroke | 1.77 | 3.81 | 6.20 | 10.15 |
| Breaststroke | 1.60 | 2.80 | 6.43 | 11.77 |
| Butterfly | 1.84 | 3.64 | 5.53 | 10.39 |
| Freestyle | 1.98 | 4.63 | 5.55 | 9.51 |
|  |  |  |  |  |
| *Males (200-m events)* |  |  |  |  |
| Backstroke | 1.62 | 3.81 | 6.46 | 10.99 |
| Breaststroke | 1.48 | 3.71 | 6.47 | 12.55 |
| Butterfly | 1.68 | 3.41 | 5.91 | 11.55 |
| Freestyle | 1.81 | 4.54 | 5.84 | 10.39 |
|  |  |  |  |  |
| *Females (100-m events)* |  |  |  |  |
| Backstroke | 1.58 | 3.11 | 7.09 | 11.48 |
| Breaststroke | 1.43 | 2.56 | 7.55 | 13.37 |
| Butterfly | 1.63 | 2.84 | 6.33 | 11.63 |
| Freestyle | 1.77 | 3.84 | 6.25 | 10.62 |
|  |  |  |  |  |
| *Females (200-m events)* |  |  |  |  |
| Backstroke | 1.48 | 3.23 | 7.61 | 12.43 |
| Breaststroke | 1.34 | 3.04 | 7.83 | 14.05 |
| Butterfly | 1.50 | 2.63 | 6.96 | 12.88 |
| Freestyle | 1.64 | 3.69 | 6.65 | 11.51 |

Appendix C.

| Appendix C. Performance benchmarks (%WR) by age category, sex and swim event derived from international elite swimmers. | | | |
| --- | --- | --- | --- |
|  | Event | Age category | Performance benchmark (%WR) |
| Males | 50 Backstroke | 18 | 111.8 |
|  | 100 Backstroke | 18 | 111.4 |
|  | 200 Backstroke | 18 | 111.0 |
|  | 50 Breaststroke | 18 | 113.0 |
|  | 100 Breaststroke | 18 | 111.5 |
|  | 200 Breaststroke | 18 | 111.8 |
|  | 50 Butterfly | 18 | 113.8 |
|  | 100 Butterfly | 18 | 111.0 |
|  | 200 Butterfly | 18 | 111.4 |
|  | 50 Freestyle | 18 | 112.9 |
|  | 100 Freestyle | 18 | 112.1 |
|  | 200 Freestyle | 18 | 111.3 |
|  | 400 Freestyle | 18 | 110.1 |
|  | 200 Medley | 18 | 111.6 |
|  | 400 Medley | 18 | 111.0 |
|  | 50 Backstroke | 19 | 111.5 |
|  | 100 Backstroke | 19 | 109.0 |
|  | 200 Backstroke | 19 | 109.5 |
|  | 50 Breaststroke | 19 | 113.0 |
|  | 100 Breaststroke | 19 | 110.9 |
|  | 200 Breaststroke | 19 | 109.4 |
|  | 50 Butterfly | 19 | 112.2 |
|  | 100 Butterfly | 19 | 109.8 |
|  | 200 Butterfly | 19 | 109.8 |
|  | 50 Freestyle | 19 | 112.7 |
|  | 100 Freestyle | 19 | 108.9 |
|  | 200 Freestyle | 19 | 110.1 |
|  | 400 Freestyle | 19 | 107.8 |
|  | 200 Medley | 19 | 110.1 |
|  | 400 Medley | 19 | 109.8 |
|  |  |  |  |
| Females | 50 Backstroke | 17 | 111.5 |
|  | 100 Backstroke | 17 | 112.1 |
|  | 200 Backstroke | 17 | 112.8 |
|  | 50 Breaststroke | 17 | 114.7 |
|  | 100 Breaststroke | 17 | 113.2 |
|  | 200 Breaststroke | 17 | 114.5 |
|  | 50 Butterfly | 17 | 116.7 |
|  | 100 Butterfly | 17 | 115.0 |
|  | 200 Butterfly | 17 | 111.6 |
|  | 50 Freestyle | 17 | 114.9 |
|  | 100 Freestyle | 17 | 110.9 |
|  | 200 Freestyle | 17 | 109.4 |
|  | 400 Freestyle | 17 | 110.8 |
|  | 200 Medley | 17 | 112.0 |
|  | 400 Medley | 17 | 111.8 |
|  | 50 Backstroke | 18 | 111.3 |
|  | 100 Backstroke | 18 | 111.1 |
|  | 200 Backstroke | 18 | 110.9 |
|  | 50 Breaststroke | 18 | 114.3 |
|  | 100 Breaststroke | 18 | 112.8 |
|  | 200 Breaststroke | 18 | 114.0 |
|  | 50 Butterfly | 18 | 117.4 |
|  | 100 Butterfly | 18 | 113.7 |
|  | 200 Butterfly | 18 | 111.2 |
|  | 50 Freestyle | 18 | 113.9 |
|  | 100 Freestyle | 18 | 111.1 |
|  | 200 Freestyle | 18 | 109.1 |
|  | 400 Freestyle | 18 | 109.6 |
|  | 200 Medley | 18 | 110.8 |
|  | 400 Medley | 18 | 110.9 |
